# 1-(4-Nitro­benzo­yl)-3-(4-nitro­phen­yl)­thio­urea acetone hemisolvate

**DOI:** 10.1107/S160053680803359X

**Published:** 2008-10-18

**Authors:** Liang Xian, Lujuan Cui, Ming Cheng

**Affiliations:** a Chemical Engineering Institute, Northwest University for Nationalities, Lanzhou 730124, People’s Republic of China

## Abstract

In the title compound, C_14_H_10_N_4_O_5_S·0.5C_3_H_6_O, the nitro­benzoyl and nitro­phenyl groups have *trans* and *cis* configurations, respectively, with respect to the thio­urea S atom. The mol­ecular conformation is stabilized by intra­molecular N—H⋯O and C—H⋯S hydrogen bonds. The acetone solvent mol­ecule possesses a crystallographically imposed twofold axis. In the crystal packing, thio­urea mol­ecules are linked by inter­molecular C—H⋯O hydrogen-bond inter­actions to form chains running parallel to the *c* axis. The chains are further bridged *via* N—H⋯O and C—H⋯O hydrogen bonds involving the acetone mol­ecules.

## Related literature

For general background on the chemistry of thio­urea derivatives, see: Choi *et al.* (2008 ▶); Jones *et al.* (2008 ▶); Kushwaha *et al.* (2008 ▶); Su *et al.* (2006 ▶). For related structures, see: Su (2005 ▶, 2007 ▶). For graph-set notation, see: Bernstein *et al.* (1995 ▶).
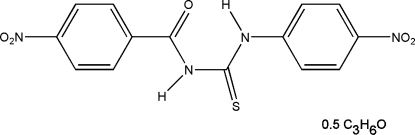


## Experimental

### 

#### Crystal data

C_14_H_10_N_4_O_5_S·0.5C_3_H_6_O
*M* *_r_* = 375.36
Monoclinic, 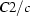

*a* = 30.828 (14) Å
*b* = 7.534 (3) Å
*c* = 15.224 (7) Å
β = 107.262 (12)°
*V* = 3377 (3) Å^3^
*Z* = 8
Mo *K*α radiation
μ = 0.23 mm^−1^
*T* = 296 (2) K
0.34 × 0.31 × 0.27 mm

#### Data collection

Bruker SMART CCD area-detector diffractometer
Absorption correction: multi-scan (*SADABS*; Sheldrick, 1996 ▶) *T*_min_ = 0.924, *T*_max_ = 0.941
9659 measured reflections
3926 independent reflections
2804 reflections with *I* > 2σ(*I*)
*R*_int_ = 0.023

#### Refinement

*R*[*F* ^2^ > 2σ(*F* ^2^)] = 0.043
*wR*(*F* ^2^) = 0.133
*S* = 1.05
3926 reflections
245 parameters
H atoms treated by a mixture of independent and constrained refinement
Δρ_max_ = 0.27 e Å^−3^
Δρ_min_ = −0.28 e Å^−3^

### 

Data collection: *SMART* (Bruker, 1998 ▶); cell refinement: *SAINT* (Bruker, 1998 ▶); data reduction: *SAINT*; program(s) used to solve structure: *SHELXS97* (Sheldrick, 2008 ▶); program(s) used to refine structure: *SHELXL97* (Sheldrick, 2008 ▶); molecular graphics: *SHELXTL* (Sheldrick, 2008 ▶); software used to prepare material for publication: *SHELXTL*.

## Supplementary Material

Crystal structure: contains datablocks global, I. DOI: 10.1107/S160053680803359X/rz2253sup1.cif

Structure factors: contains datablocks I. DOI: 10.1107/S160053680803359X/rz2253Isup2.hkl

Additional supplementary materials:  crystallographic information; 3D view; checkCIF report

## Figures and Tables

**Table 1:** Hydrogen-bond geometry (Å, °)

*D*—H⋯*A*	*D*—H	H⋯*A*	*D*⋯*A*	*D*—H⋯*A*
C1—H1⋯S1	0.93	2.79	3.235 (2)	111
N2—H2*A*⋯O3	0.90 (3)	1.88 (3)	2.659 (3)	144 (2)
C2—H2⋯O4^i^	0.93	2.48	3.394 (3)	167
C12—H12⋯O5^ii^	0.93	2.54	3.318 (3)	141
N3—H3*A*⋯O6	0.86 (2)	2.442 (19)	3.300 (2)	175 (2)
C13—H13⋯O6	0.93	2.59	3.207 (3)	124
C14—H14*B*⋯O5^iii^	0.96	2.56	3.446 (3)	154
C14—H14*C*⋯O4^iv^	0.96	2.52	3.476 (3)	175

Symmetry codes: (i) 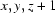; (ii) 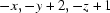; (iii) 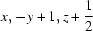; (iv) 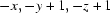.

**Fig. 1.**
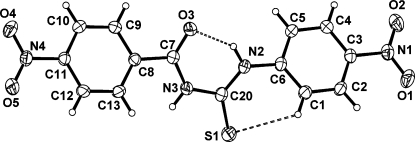
The molecular structure of the title compound. Displacement ellipsoids are drawn at the 30% probability level. Intramolecular hydrogen bonds are shown as dashed lines.

**Fig. 2.**
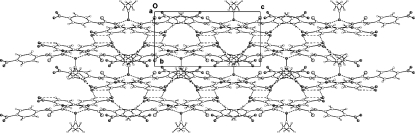
Packing diagram of the title compound viewed along the a axis. Intermolecular hydrogen bonds are shown as dashed lines.

## supplementary crystallographic information

### Comment

Thiourea and its derivatives are broadly applied in anion recognition, nonlinear optics and catalysis, and display also high bioactivity and good coordination ability (Choi *et al.*, 2008; Kushwaha *et al.*, 2008; Jones *et al.* 2008; Su *et al.*, 2006). As part of our research on thiourea coordination chemistry, we are interested in the study of the influence of noncovalent interactions, especially hydrogen bonds and π-π stacking interactions, on the coordination modes of benzoylthiourea with transition metal ions. In the present paper, the crystal structure of the title compound is reported.

In the molecule of the title compound (Fig. 1), the nitrobenzoyl and nitrophenyl groups have *trans* and *cis* configurations, respectively, with respect of the thiourea S atom. The dihedral angle formed by the two aromatic rings is 7.68 (6)°. The molecular conformation is stabilized by intramolecular N—H···O and C—H···S hydrogen bonds (Table 1) forming six-membered rings of graph set *S*(6) (Bernstein *et al.*, 1995). This conformation is similar to that reported for N-(4-chlorophenyl)-N'-(4-nitrobenzoyl)urea (Su, 2005) and for N-(p-nitrobenzoyl)-N'-(p-chlorophenyl)thiourea (Su, 2007). The acetone solvent molecule has a crystallographically imposed twofold symmetry. In the crystal packing (Fig. 2), thiourea molecules are linked into chains running parallel to the *c* axis by intermolecular C—H···O hydrogen bonds (Table 1). These chains are further bridged *via* N—H···O and C—H···O hydrogen bonds (Table 1) involving the acetone molecules.

### Experimental

All reagents and organic solvents were of analytical reagent grade and commercially available. p-Nitrobenzoyl chloride (1.86 g) was treated with ammonium thiocyanate (1.20 g) in CH_2_Cl_2_ under solid-liquid phase transfer catalysis conditions, using 3% polyethylene glycol-400 as catalyst, to give the corresponding benzoyl isothiocyanate, which was reacted with p-nitroaniline (1.38 g) to give the title compound. The solid was separated from the liquid phase by filtration, washed with CH_2_Cl_2_ and then dried in air. Yellow single crystals suitable for X-ray analysis were obtained after one week by slow evaporation of an acetone solution. The infrared spectrum was recorded in the range of 4000–400 cm^-1^ on a Nicolet NEXUS 670 F T—IR spectrometer, using KBr pellets. ^1^H NMR spectrum was obtained on an INOVA-400 MHz superconduction spectrometer, DMSO-_d6_ was used as solvent and TMS as internal standard, and the chemical shifts are expressed as delta. Elemental analyses were carried out on a PE-2400 elemental analysis instrument. Melting point determination was performed in YRT-3 melting point instrument (Tianjin) and was uncorrected. Melting Point: 201–204°C. Elemental analysis (%) found (calcd.): C, 50.25(49.6); H, 3.55(3.47); N, 11.30(14.93); S, 8.50(8.53). IR (KBr, cm^-1^): 3385 (N—H), 3064, 1680 (C=O), 1571(C=C), 1318, 1259(C=S), 1137, 1106. ^1^H NMR(delta, p.p.m.): 2.50 (s, 6H, 2CH_3_); 8.07–8.38 (m, 8H, 2C_6_H_4_); 12.15 (s, 1H, NH); 12.61 (s, 1H, NH).

### Refinement

All H atoms bound to C atoms were placed in calculated positions and refined using the riding model approximation, with C—H = 0.93-0.96 Å and with *U*_iso_(H) = 1.2*U*_eq_(C) or 1.5 *U*_eq_(C) for methyl H atoms. The H atoms bound to N atoms were located in a difference Fourier map and refined freely.

### Crystal data

**Table:**

C_14_H_10_N_4_O_5_S·0.5C_3_H_6_O	*F*(000) = 1552
*M**_r_* = 375.36	*D*_x_ = 1.477 Mg m^−^^3^
Monoclinic, *C*2/*c*	Mo *K*α radiation, λ = 0.71073 Å
Hall symbol: -C 2yc	Cell parameters from 3344 reflections
*a* = 30.828 (14) Å	θ = 2.7–28.3°
*b* = 7.534 (3) Å	µ = 0.23 mm^−^^1^
*c* = 15.224 (7) Å	*T* = 296 K
β = 107.262 (12)°	Block, yellow
*V* = 3377 (3) Å^3^	0.34 × 0.31 × 0.27 mm
*Z* = 8	

### Data collection

**Table:**

Bruker SMART CCD area-detector diffractometer	3926 independent reflections
Radiation source: fine-focus sealed tube	2804 reflections with *I* > 2σ(*I*)
graphite	*R*_int_ = 0.023
φ and ω scans	θ_max_ = 28.0°, θ_min_ = 1.4°
Absorption correction: multi-scan (SADABS; Sheldrick, 1996)	*h* = −39→32
*T*_min_ = 0.924, *T*_max_ = 0.941	*k* = −9→9
9659 measured reflections	*l* = −19→19

### Refinement

**Table:**

Refinement on *F*^2^	Primary atom site location: structure-invariant direct methods
Least-squares matrix: full	Secondary atom site location: difference Fourier map
*R*[*F*^2^ > 2σ(*F*^2^)] = 0.043	Hydrogen site location: inferred from neighbouring sites
*wR*(*F*^2^) = 0.133	H atoms treated by a mixture of independent and constrained refinement
*S* = 1.05	*w* = 1/[σ^2^(*F*_o_^2^) + (0.063*P*)^2^ + 1.6044*P*] where *P* = (*F*_o_^2^ + 2*F*_c_^2^)/3
3926 reflections	(Δ/σ)_max_ = 0.001
245 parameters	Δρ_max_ = 0.27 e Å^−^^3^
0 restraints	Δρ_min_ = −0.28 e Å^−^^3^

### Special details

**Table:**

Geometry. All e.s.d.'s (except the e.s.d. in the dihedral angle between two l.s. planes) are estimated using the full covariance matrix. The cell e.s.d.'s are taken into account individually in the estimation of e.s.d.'s in distances, angles and torsion angles; correlations between e.s.d.'s in cell parameters are only used when they are defined by crystal symmetry. An approximate (isotropic) treatment of cell e.s.d.'s is used for estimating e.s.d.'s involving l.s. planes.
Refinement. Refinement of *F*^2^ against ALL reflections. The weighted *R*-factor *wR* and goodness of fit *S* are based on *F*^2^, conventional *R*-factors *R* are based on *F*, with *F* set to zero for negative *F*^2^. The threshold expression of *F*^2^ > σ(*F*^2^) is used only for calculating *R*-factors(gt) *etc*. and is not relevant to the choice of reflections for refinement. *R*-factors based on *F*^2^ are statistically about twice as large as those based on *F*, and *R*- factors based on ALL data will be even larger.

### Fractional atomic coordinates and isotropic or equivalent isotropic displacement parameters (Å^2^)

**Table:**

	*x*	*y*	*z*	*U*_iso_*/*U*_eq_	
S1	0.06837 (2)	0.61209 (9)	0.99931 (4)	0.0607 (2)	
N2	0.15507 (6)	0.7121 (2)	1.02255 (11)	0.0464 (4)	
C8	0.11880 (6)	0.8073 (2)	0.72951 (12)	0.0378 (4)	
O3	0.17768 (5)	0.7877 (2)	0.87122 (9)	0.0576 (4)	
C7	0.13716 (6)	0.7739 (3)	0.83134 (12)	0.0418 (4)	
C11	0.09121 (6)	0.8598 (2)	0.54287 (12)	0.0393 (4)	
C3	0.21046 (6)	0.6165 (2)	1.30383 (12)	0.0406 (4)	
N4	0.07617 (6)	0.8875 (2)	0.44189 (11)	0.0494 (4)	
N1	0.22987 (6)	0.5784 (2)	1.40277 (11)	0.0501 (4)	
N3	0.10573 (6)	0.7234 (2)	0.87465 (11)	0.0443 (4)	
C6	0.17262 (6)	0.6799 (2)	1.11874 (12)	0.0402 (4)	
O4	0.10416 (6)	0.8676 (3)	0.39985 (10)	0.0737 (5)	
C12	0.06084 (7)	0.8910 (3)	0.59207 (13)	0.0473 (5)	
H12	0.0315	0.9292	0.5624	0.057*	
O1	0.20702 (6)	0.6125 (2)	1.45421 (11)	0.0714 (5)	
C10	0.13499 (7)	0.8041 (3)	0.58343 (13)	0.0450 (5)	
H10	0.1548	0.7844	0.5487	0.054*	
C5	0.21595 (7)	0.6070 (3)	1.15057 (13)	0.0440 (5)	
H5	0.2322	0.5810	1.1095	0.053*	
C4	0.23498 (6)	0.5731 (3)	1.24383 (13)	0.0433 (4)	
H4	0.2637	0.5222	1.2656	0.052*	
C13	0.07482 (6)	0.8645 (3)	0.68621 (13)	0.0455 (5)	
H13	0.0548	0.8850	0.7204	0.055*	
C9	0.14873 (6)	0.7782 (3)	0.67771 (13)	0.0434 (4)	
H9	0.1783	0.7409	0.7069	0.052*	
C2	0.16818 (7)	0.6953 (3)	1.27367 (13)	0.0467 (5)	
H2	0.1527	0.7270	1.3153	0.056*	
C20	0.11244 (7)	0.6852 (3)	0.96836 (13)	0.0420 (4)	
C1	0.14910 (7)	0.7265 (3)	1.18041 (13)	0.0479 (5)	
H1	0.1206	0.7787	1.1591	0.057*	
O2	0.26788 (6)	0.5125 (3)	1.42899 (10)	0.0722 (5)	
O5	0.03714 (6)	0.9316 (3)	0.40451 (10)	0.0722 (5)	
O6	0.0000	0.6468 (3)	0.7500	0.0792 (8)	
C15	0.0000	0.4863 (4)	0.7500	0.0500 (7)	
C14	−0.03535 (9)	0.3861 (3)	0.7781 (2)	0.0754 (7)	
H14A	−0.0541	0.4678	0.7990	0.113*	
H14B	−0.0210	0.3057	0.8270	0.113*	
H14C	−0.0538	0.3203	0.7265	0.113*	
H3A	0.0780 (7)	0.711 (3)	0.8413 (14)	0.049 (6)*	
H2A	0.1744 (9)	0.733 (4)	0.9897 (18)	0.081 (8)*	

### Atomic displacement parameters (Å^2^)

**Table:**

	*U*^11^	*U*^22^	*U*^33^	*U*^12^	*U*^13^	*U*^23^
S1	0.0563 (4)	0.0823 (4)	0.0462 (3)	−0.0144 (3)	0.0194 (3)	0.0088 (3)
N2	0.0452 (10)	0.0642 (11)	0.0310 (8)	−0.0001 (8)	0.0131 (7)	0.0046 (7)
C8	0.0392 (10)	0.0399 (10)	0.0344 (9)	−0.0020 (7)	0.0112 (7)	0.0026 (7)
O3	0.0408 (8)	0.0929 (12)	0.0379 (7)	−0.0029 (7)	0.0096 (6)	0.0058 (7)
C7	0.0420 (10)	0.0464 (11)	0.0371 (10)	−0.0002 (8)	0.0122 (8)	0.0013 (8)
C11	0.0419 (10)	0.0420 (10)	0.0339 (9)	−0.0017 (8)	0.0111 (8)	0.0008 (7)
C3	0.0459 (11)	0.0421 (10)	0.0321 (9)	−0.0038 (8)	0.0091 (8)	0.0008 (7)
N4	0.0564 (11)	0.0560 (11)	0.0353 (9)	0.0027 (8)	0.0126 (8)	−0.0015 (7)
N1	0.0566 (11)	0.0545 (10)	0.0368 (9)	−0.0028 (8)	0.0099 (8)	0.0020 (7)
N3	0.0400 (9)	0.0574 (10)	0.0340 (8)	−0.0039 (8)	0.0089 (7)	0.0049 (7)
C6	0.0458 (10)	0.0433 (10)	0.0317 (9)	−0.0013 (8)	0.0119 (8)	0.0012 (8)
O4	0.0754 (12)	0.1094 (15)	0.0437 (9)	0.0136 (10)	0.0289 (8)	0.0056 (9)
C12	0.0370 (10)	0.0629 (13)	0.0415 (10)	0.0084 (9)	0.0108 (8)	0.0091 (9)
O1	0.0845 (12)	0.0950 (13)	0.0394 (8)	0.0095 (10)	0.0257 (8)	0.0075 (8)
C10	0.0427 (11)	0.0536 (12)	0.0426 (10)	0.0032 (9)	0.0189 (8)	−0.0015 (9)
C5	0.0419 (10)	0.0553 (12)	0.0379 (10)	−0.0018 (8)	0.0164 (8)	−0.0049 (8)
C4	0.0382 (10)	0.0493 (11)	0.0411 (10)	0.0002 (8)	0.0096 (8)	−0.0024 (8)
C13	0.0395 (10)	0.0596 (12)	0.0415 (10)	0.0052 (9)	0.0184 (8)	0.0060 (9)
C9	0.0351 (10)	0.0529 (12)	0.0415 (10)	0.0047 (8)	0.0101 (8)	0.0021 (8)
C2	0.0505 (12)	0.0576 (12)	0.0359 (10)	0.0067 (9)	0.0187 (8)	0.0008 (9)
C20	0.0487 (11)	0.0420 (10)	0.0363 (10)	0.0010 (8)	0.0138 (8)	0.0027 (8)
C1	0.0477 (11)	0.0570 (12)	0.0390 (10)	0.0132 (9)	0.0130 (8)	0.0037 (9)
O2	0.0641 (10)	0.0925 (13)	0.0481 (9)	0.0169 (9)	−0.0016 (7)	0.0059 (9)
O5	0.0597 (10)	0.1078 (14)	0.0417 (8)	0.0188 (9)	0.0038 (7)	0.0013 (9)
O6	0.0867 (19)	0.0590 (15)	0.111 (2)	0.000	0.0586 (17)	0.000
C15	0.0476 (16)	0.060 (2)	0.0417 (15)	0.000	0.0121 (12)	0.000
C14	0.0655 (16)	0.0731 (17)	0.096 (2)	−0.0050 (13)	0.0367 (15)	0.0070 (14)

### Geometric parameters (Å, °)

**Table:**

S1—C20	1.659 (2)	N3—H3A	0.86 (2)
N2—C20	1.344 (3)	C6—C1	1.391 (3)
N2—C6	1.423 (2)	C6—C5	1.392 (3)
N2—H2A	0.90 (3)	C12—C13	1.383 (3)
C8—C13	1.389 (3)	C12—H12	0.9300
C8—C9	1.397 (3)	C10—C9	1.385 (3)
C8—C7	1.505 (3)	C10—H10	0.9300
O3—C7	1.221 (2)	C5—C4	1.389 (3)
C7—N3	1.378 (2)	C5—H5	0.9300
C11—C10	1.373 (3)	C4—H4	0.9300
C11—C12	1.382 (3)	C13—H13	0.9300
C11—N4	1.483 (2)	C9—H9	0.9300
C3—C2	1.381 (3)	C2—C1	1.386 (3)
C3—C4	1.387 (3)	C2—H2	0.9300
C3—N1	1.475 (2)	C1—H1	0.9300
N4—O5	1.215 (2)	O6—C15	1.209 (4)
N4—O4	1.227 (2)	C15—C14	1.489 (3)
N1—O2	1.225 (2)	C14—H14A	0.9600
N1—O1	1.226 (2)	C14—H14B	0.9600
N3—C20	1.409 (2)	C14—H14C	0.9600
			
C20—N2—C6	127.59 (17)	C11—C10—H10	121.1
C20—N2—H2A	112.0 (17)	C9—C10—H10	121.1
C6—N2—H2A	119.4 (17)	C4—C5—C6	119.93 (17)
C13—C8—C9	119.73 (17)	C4—C5—H5	120.0
C13—C8—C7	123.80 (16)	C6—C5—H5	120.0
C9—C8—C7	116.46 (16)	C3—C4—C5	118.84 (18)
O3—C7—N3	123.16 (17)	C3—C4—H4	120.6
O3—C7—C8	120.96 (16)	C5—C4—H4	120.6
N3—C7—C8	115.86 (16)	C12—C13—C8	119.79 (17)
C10—C11—C12	122.83 (17)	C12—C13—H13	120.1
C10—C11—N4	118.20 (16)	C8—C13—H13	120.1
C12—C11—N4	118.96 (17)	C10—C9—C8	120.85 (17)
C2—C3—C4	121.85 (17)	C10—C9—H9	119.6
C2—C3—N1	118.69 (17)	C8—C9—H9	119.6
C4—C3—N1	119.46 (18)	C3—C2—C1	119.02 (18)
O5—N4—O4	122.79 (18)	C3—C2—H2	120.5
O5—N4—C11	119.06 (17)	C1—C2—H2	120.5
O4—N4—C11	118.14 (17)	N2—C20—N3	114.45 (16)
O2—N1—O1	123.50 (18)	N2—C20—S1	127.59 (15)
O2—N1—C3	118.07 (17)	N3—C20—S1	117.96 (15)
O1—N1—C3	118.43 (18)	C2—C1—C6	120.06 (18)
C7—N3—C20	128.92 (17)	C2—C1—H1	120.0
C7—N3—H3A	117.7 (14)	C6—C1—H1	120.0
C20—N3—H3A	113.4 (14)	O6—C15—C14	120.45 (15)
C1—C6—C5	120.22 (17)	C15—C14—H14A	109.5
C1—C6—N2	122.44 (18)	C15—C14—H14B	109.5
C5—C6—N2	117.26 (16)	H14A—C14—H14B	109.5
C11—C12—C13	118.96 (17)	C15—C14—H14C	109.5
C11—C12—H12	120.5	H14A—C14—H14C	109.5
C13—C12—H12	120.5	H14B—C14—H14C	109.5
C11—C10—C9	117.83 (17)		
			
C13—C8—C7—O3	−152.9 (2)	C1—C6—C5—C4	2.9 (3)
C9—C8—C7—O3	26.9 (3)	N2—C6—C5—C4	179.80 (17)
C13—C8—C7—N3	28.7 (3)	C2—C3—C4—C5	−1.2 (3)
C9—C8—C7—N3	−151.55 (18)	N1—C3—C4—C5	178.93 (17)
C10—C11—N4—O5	177.8 (2)	C6—C5—C4—C3	−1.3 (3)
C12—C11—N4—O5	−2.3 (3)	C11—C12—C13—C8	0.0 (3)
C10—C11—N4—O4	−3.4 (3)	C9—C8—C13—C12	0.5 (3)
C12—C11—N4—O4	176.51 (19)	C7—C8—C13—C12	−179.73 (18)
C2—C3—N1—O2	−178.25 (19)	C11—C10—C9—C8	0.3 (3)
C4—C3—N1—O2	1.6 (3)	C13—C8—C9—C10	−0.6 (3)
C2—C3—N1—O1	2.5 (3)	C7—C8—C9—C10	179.60 (18)
C4—C3—N1—O1	−177.63 (19)	C4—C3—C2—C1	2.1 (3)
O3—C7—N3—C20	2.4 (3)	N1—C3—C2—C1	−178.03 (18)
C8—C7—N3—C20	−179.26 (18)	C6—N2—C20—N3	−177.66 (18)
C20—N2—C6—C1	−43.5 (3)	C6—N2—C20—S1	1.7 (3)
C20—N2—C6—C5	139.8 (2)	C7—N3—C20—N2	4.1 (3)
C10—C11—C12—C13	−0.3 (3)	C7—N3—C20—S1	−175.35 (17)
N4—C11—C12—C13	179.78 (18)	C3—C2—C1—C6	−0.5 (3)
C12—C11—C10—C9	0.2 (3)	C5—C6—C1—C2	−2.0 (3)
N4—C11—C10—C9	−179.88 (17)	N2—C6—C1—C2	−178.72 (18)

### Hydrogen-bond geometry (Å, °)

**Table:**

*D*—H···*A*	*D*—H	H···*A*	*D*···*A*	*D*—H···*A*
C1—H1···S1	0.93	2.79	3.235 (2)	111
N2—H2A···O3	0.90 (3)	1.88 (3)	2.659 (3)	144 (2)
C2—H2···O4^i^	0.93	2.48	3.394 (3)	167
C12—H12···O5^ii^	0.93	2.54	3.318 (3)	141
N3—H3A···O6	0.86 (2)	2.442 (19)	3.300 (2)	175 (2)
C13—H13···O6	0.93	2.59	3.207 (3)	124
C14—H14B···O5^iii^	0.96	2.56	3.446 (3)	154
C14—H14C···O4^iv^	0.96	2.52	3.476 (3)	175

Symmetry codes: (i) *x*, *y*, *z*+1; (ii) −*x*, −*y*+2, −*z*+1; (iii) *x*, −*y*+1, *z*+1/2; (iv) −*x*, −*y*+1, −*z*+1.

## References

[bb1] Bernstein, J., Davis, R. E., Shimoni, L. & Chang, N.-L. (1995). *Angew. Chem. Int. Ed. Engl.* **34**, 1555–1573.

[bb2] Bruker (1998). *SMART* and *SAINT* Bruker AXS Inc., Madison, Wisconsin, USA.

[bb3] Choi, M. K., Kim, H. N., Choi, H. J., Yoon, J. & Hyun, M. H. (2008). *Tetrahedron Lett.* **49**, 4522–4525.

[bb4] Jones, C. E. S., Turega, S. M., Clarke, M. L. & Philp, D. (2008). *Tetrahedron Lett.* **49**, 4666–4669.

[bb5] Kushwaha, S. K., Vijayan, N. & Bhagavannarayana, G. (2008). *Mater. Lett.* **62**, 3931–3933.

[bb6] Sheldrick, G. M. (1996). *SADABS* University of Göttingen, Germany.

[bb7] Sheldrick, G. M. (2008). *Acta Cryst.* A**64**, 112–122. 10.1107/S0108767307043930 18156677

[bb8] Su, B.-Q. (2005). *Acta Cryst.* E**61**, o3492–o3494.

[bb9] Su, B.-Q. (2007). *J. Chem. Crystallogr.* **37**, 87–90.

[bb10] Su, B.-Q., Liu, G.-L., Sheng, L., Wang, X.-Q. & Xian, L. (2006). *Phosphorus Sulfur Slicon*, **181**, 745–750.

